# Non-communicable diseases in humanitarian settings: ten essential questions

**DOI:** 10.1186/s13031-017-0119-8

**Published:** 2017-09-17

**Authors:** S. Aebischer Perone, E. Martinez, S. du Mortier, R. Rossi, M. Pahud, V. Urbaniak, F. Chappuis, O. Hagon, F. Jacquérioz Bausch, D. Beran

**Affiliations:** 1 Health Unit, International Committee of the Red Cross (ICRC), 19, avenue de la Paix, 1202 Geneva, Switzerland; 2 Division of Tropical and Humanitarian Medicine, Geneva University Hospitals, Rue Gabrielle-Perret-Gentil 4, 1205 Geneva, Switzerland; 3 Division of Tropical and Humanitarian Medicine, Geneva University Hospitals, Faculty of Medicine, University of Geneva, Rue Gabrielle-Perret-Gentil 6, 1205 Geneva, Switzerland

**Keywords:** Non-communicable diseases, Chronic diseases, Conflicts, Crises, Humanitarian emergencies, Continuum of care, Ethics, Humanitarian agencies

## Abstract

Non-communicable diseases (NCDs) represent the primary cause of morbidity and mortality worldwide. Specific attention needs to be given in fragile and crisis-affected contexts, where health systems have even more difficulties in addressing and managing these diseases. Humanitarian actors intervening in crisis situations increasingly include NCD management in the services they support and provide. This review aims at presenting a series of questions that humanitarian agencies could consider when addressing NCDs in humanitarian crises. They include, among others, what conditions to address and for which target population, how to ensure continuity of care, which guidelines and medications to use, and what can be done beyond classical management of NCDs.

Research and evidence are lacking on how to address care effectively for NCDs in emergencies. Therefore, advocacy is needed for NCD-oriented research so as to make interventions more effective and sustainable. No government or single agency can address NCDs in humanitarian crises alone. Strong leadership and partnerships between humanitarian actors, health providers, government bodies, research and academic institutions are required. Only a coordinated multi-disciplinary and multi-stakeholder approach will achieve the required impact for affected populations.

## Background

**Fig 1:**
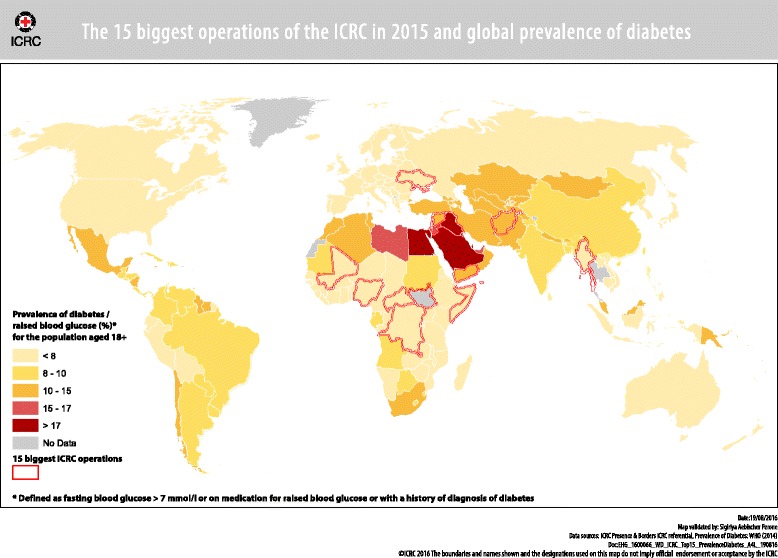
The 15 biggest operations of the ICRC in 2015 and global prevalence of diabetes. Prevalence of diabetes / raised blood glucose (%) for the population aged 18+; Prevalence of diabetes: WHO: 2014 15 biggest ICRC operations in 2015

Non-communicable diseases, (NCDs), are the leading cause of mortality worldwide with 38 million deaths, (68%), mainly due to cardiovascular diseases, diabetes, chronic respiratory diseases and cancer [1]. Nearly three quarters of NCD-related deaths, (28 million), occur in low and middle-income countries, (LMICs). In addition, in many LMICs, the burden of NCDs is concurrent to the burden of infectious diseases causing a double burden of disease and stretching the capacities of weak health care systems [2]. In more than half of the countries where the International Committee of the Red Cross, (ICRC), conducts its main operations, the prevalence of diabetes among the population above 18 years is higher than 10%. *(* *Fig.* *1* *: Map with 15 main ICRC operations and distribution of diabetes).* The epidemiological transition with a change from infectious to non-communicable diseases has obliged the ICRC over time to adjust its medical response to increasing needs, commencing in the Balkans, followed by Lebanon, Pakistan, Liberia and Sudan in 2004 and later Yemen, Syria and Iraq and countries of the region that receive refugees [3]. In those countries, diabetes is the cause of more than 25% of amputations in 1/3 of the centres in the cohort of amputees in ICRC’s Physical Rehabilitation Centres [4].

Humanitarian emergencies or crises can result from internal or external conflicts, natural disasters, epidemic or pandemic diseases, and complex emergencies [5]*.* Levels range from an emerging crisis, (essential needs may or may not be covered), to an acute crisis, (or emergency phase of a crisis where some basic needs are no longer covered), a chronic crisis (insufficient coverage of basic needs, a return to an acute crisis is possible), and post-crisis, (essential needs are covered by structures whose viability remains fragile) [6]. These emergencies cause disruption of access to existing resources and further impair the capacity of services to meet essential needs due to the break down in authority, health care systems, and/or societal cohesiveness.

The aim of the humanitarian response is to use a public health approach when assisting persons affected by a crisis. In emergencies, the priority is to minimise mortality as far as possible and to address the most urgent needs. The scope of the response depends on the type and phase of the crisis, the number of affected people and the pre-existing burden of disease and fragility.

LMICs are affected disproportionally by humanitarian crises, often secondary to armed conflict. In these contexts, already fragile health care systems are often not able to respond to the sudden increase in acute and chronic care needs in a timely and proper manner [7]*.* For the population, in addition to the risk of acute illnesses directly related to the crisis, the risk of exacerbating pre-existing chronic conditions, and suffering from NCD complications, increases substantially [8, 9].

Besides the direct impact of armed conflict on the health status of the population, (due to death, injury and disability), there are also indirect effects, such as displacement, economic loss, and psychological consequences that further aggravate the situation. Humanitarian emergencies at large put pressure on health systems, which, in order to cope with urgent requirements, often have to neglect the management of chronic diseases [10]. Hayman et al. [11] found that acute NCD complications, such as myocardial infarction, were higher in conflict settings and after natural disasters than in pre-emergency circumstances.

In natural disasters the pattern of disease greatly differs according to the nature of the disaster. For example, there are differences in morbidity and mortality between an earthquake and a tsunami with a higher prevalence of severe injuries in an earthquake [12], as well as between rural or urban areas. Trauma care overburdens health systems in natural disasters essentially at their onset [13] in addition to underlying burden of NCDs requiring continuous care [14–16].

Health systems might be disrupted as a consequence of the disasters, irrespective of resulting pattern of disease, or not being able to face the number of casualties. In such cases the reallocation of resources to cope with increased urgent demands might pose challenges to the continuity of care for persons with NCDs [17].

In addition, different populations have different needs. While all persons affected by a conflict endure the disruption of routine health activities [10, 18] and are exposed to overwhelmed, under-equipped and under-staffed health care services [19], people directly affected by conflict, and still living in conflict zones face additionally the risk of violence from the conflict thereby causing an extra burden on the health system with need of emergency surgical care. Today, moreover, a higher number of internally displaced persons, (IDPs), and refugees are living in urban settings rather than in camps [20]*.* This requires humanitarian agencies to adapt their traditional response in order to provide optimal interventions for NCDs. For urban settings, wherever possible the existing Ministry of Health, (MoH), primary care health structures should be reinforced with human and material resources so as to increase their capacity. However in camps, humanitarian organisations may need to establish ad hoc primary care health structures.

Included in the WHO’s NCD Global Action Plan (GAP) are the following statements regarding NCDs and the humanitarian response: [21]*.*

“Improve the availability of life-saving technologies and essential medicines for managing NCDs in the initial phase of emergency response.”

“It must be ensured that the use of these services does not expose the users to financial hardship, including in cases of ensuring the continuity of care in the aftermath of emergencies and disasters.”

The GAP also prioritises cardiovascular diseases, diabetes, cancer and chronic respiratory diseases, as being the main NCDs that the global health community needs to address.

Despite these statements, NCDs to date have not received the necessary attention in emergency contexts [9]*.* Although humanitarian actors have started including NCDs in their programming, the challenges in emergency settings abound. These chronic conditions require approaches that are different from acute condition approaches, and often demand long term commitment [22].

Every humanitarian emergency and setting has its specific needs and priorities. Need in NCD care is present in both Syrian refugees in Jordan [23], and in sub-Saharan settings, (although different). In addition, Mali for example, NCDs have not been included in either an annual global response plan or to an emergency response, thereby creating potentially fatal consequences for patients [24]. Nevertheless, there are underlying commonalities which need to be addressed. This review therefore aims at presenting ten fundamental questions that humanitarian agencies should consider when addressing NCDs in humanitarian crises. The focus being on acute and chronic crises, (see Table 1). Given that humanitarian response includes emergency interventions, (acute crisis), as well as long-term response, (chronic crisis), answering the ten questions needs to be repeated when moving from one level to the other. Different weight is given to each question depending on the level of the crisis with larger scope in a chronic crisis.

**Table 1:** Ten essential questions for developing a humanitarian response to NCDs

1. What are the existing capacities of the local health system?	
2. Which NCDs to address?	
3. Who is the target population?	
4. What kind of interventions needed to ensure continuum and continuity of care?	
5. Which algorithms or guidelines to use?	
6. What medications to be integrated in the basic essential drug list?	
7. What are the ethical implications?	
8. How to ensure accountability to patients?	
9. How to monitor interventions?	
10. What to do beyond provision of health services for “classical” NCDs?	

## Main text

### What are the existing capacities of the local health system?

The level of pre-crisis care for NCDs and remaining capacity of the existing health system needs to be assessed before considering any intervention in a humanitarian emergency [25]*.* This allows to prioritise where and how to support best and tailor the response to the needs. Elements requiring assessment: health care structure, level at which NCDs are addressed, disease related policies and protocols, programmes and activities, human resources at primary and secondary health care levels, (allocation and training), material resources, (supply and procurement system, price and affordability of care), access to care, referral mechanisms, patients’ and families’ perspectives and links with communities and community agencies.

A crisis places strain on potentially already weak local health systems. The extra burden of NCDs on remaining health facilities is secondary to the provision of care to larger populations, (e.g. IDPs and refugees), the exacerbation of pre-existing chronic conditions and the destruction of health facilities and/or health care workers fleeing conflict zones. [10, 26, 27]. Interventions for NCDs in public health emergencies, such as Ebola or SARS outbreaks, face such challenges. For instance, during the 2014 Ebola outbreak in West Africa, NCD care and programmes were among the first to be interrupted amid lack of human and financial resources and closure of routine health services, (*personal communication, Jacquerioz Bausch F 2016*) [28]. Many health care workers fled the workplace due to fear of becoming infected. Patients were not attending facilities because they were afraid of being contaminated [29]. Systematic screening, infection prevention and control measures, and provision of appropriate personal protective equipment for health care workers, eventually allowed the restoration of basic non-Ebola health services in parallel with the establishment of Ebola treatment centres.

In a crisis situation, allocation of resources by the authorities, including human resources, is influenced by data related to the needs [30]*,* preparedness of the health system for NCD management in emergencies, willingness and readiness to address NCDs, need for training [31]*,* types of activities and support offered by humanitarian organizations and acceptance of task-shifting by the authorities to a lower level of care under supervision. When designing an answer to NCDs in emergencies, leadership, policy and financial issues need to be addressed and coordinated by all actors involved, taking into account emergency and continuing response [8, 27].

In fragile contexts, reliable pre-crisis data on burden of disease is rarely available [32], resulting in the need to develop, test and validate NCD-specific questions in assessment tools [33]*.* To date, humanitarian organizations do not have a standard rapid assessment tool for NCDs, which is needed for decision-making [34]. The Service Availability and Readiness Assessment (SARA) tool of the WHO [25] gives a global overview on existing services for NCDs, but this needs to be completed with baseline and routine data for NCDs from national governments, as well as data collected from health information system databases [31]. Rapid assessment protocols for insulin access, (RAPIA), have been used in resource-limited countries to address diabetes and could be used for other NCDs [35] and be adapted and simplified for humanitarian settings.

### Which NCD to address?

The choice of prioritising NCDs to be addressed in an emergency is informed by the assessment of the existing health system, (pre-crisis burden of disease and availability of care, see # 1), disease prognosis in cases of treatment interruption, feasibility of treatment plan and follow-up care, the level and type of crisis, and the responder’s capacity to provide sustainable and cost-effective care. While lifesaving care is prioritized in the first response to an acute crisis, whatever its origin, it is essential to consider the sustainability of an intervention and handover to local health authorities/structures in a chronic crisis, (e.g. specialised organisations supporting existing haemodialysis).

Other considerations include drug costs and availability of human and financial resources [36].

The WHO has prioritised cardiovascular diseases, (i.e ischemic heart disease, high blood pressure), cancers, diabetes and chronic respiratory diseases, (i.e asthma, chronic obstructive pulmonary disease) for global public health response in general, rather than their importance in emergencies. Based on the above considerations, cancer is a good example of an NCD that has to be further discussed in terms of relevance and feasibility. Care, except for pain management and symptom relief, might be complex, expensive and rarely achievable amidst humanitarian crises. Depending on the assessment of the local health system, diseases like type 1 diabetes, where treatment interruption causes rapid decompensations, should be prioritised. Médecins Sans Frontières, (MSF) [37, 38], the ICRC, the United Nations Relief and Works Agency for Palestine Refugees in the Near East (UNWRA) [39, 40], and the United Nations High Commissioner for Refugees (UNHCR) [41, 42] covering the needs of refugees in the Near and Middle East are currently prioritising cardiovascular diseases with hypertension, diabetes and chronic respiratory diseases. Inclusion of other chronic conditions such as mental health disorders and epilepsy should also be considered when designing an intervention based on local prevalence and relevance, (see question # 10).

### Who is the target population for health interventions?

Stressors and protective factors differ between groups of population affected by humanitarian emergencies, (e.g. displaced/host population and within those groups: women, children, elderly, handicapped…) [43], and a variety of actors is warranted to address their health needs [19]. When establishing health services in a humanitarian context, it is essential to respect the principle of equity. Resident or host populations should have access to similar services as IDPs and refugees [30]. Reinforcement of the public health sector benefits both parties [44]. Refugees and IDPs living outside areas covered by any functioning health system could benefit from mobile medical teams visiting remote areas on a regular basis, as provided under the guidance of the MoH in Northern Iraq [45]. Access to care can also be improved by providing vouchers to patients for consultations, (when health care structures are in place), or organising transport to health care centres, as done for example by the ICRC in Afghanistan for war wounded. Nomadic people and persons with difficult access to health care centres, due to transport challenges or security reasons, could also be provided with monthly treatment kits.

Moreover, NCD interventions need to target individuals with a range of behavioural and biological risk factors and diseases, in addition to possible crisis-related injuries and mental health issues, (either existing prior to humanitarian crisis, or caused by the crisis). Persons affected by NCDs and presenting “classical” and mental health risk factors may need closer follow-up care than those without. Risk factors in crisis are therefore considered to categorise risk and set priorities of care, with limited possibility of reducing the risk factors during the acute phase of a crisis. (Table 2).

**Table 2:** Table adapted from Beran 2015 – Karger Chapter [87]

	Risk factor	NCD	Mental Health	
			Prior to crisis	Crisis related
Risk factor	Person who smokes and is obese	Person with diabetes who smokes	Smoking and alcohol consumption	Smoking and alcohol consumption as a coping mechanism
NCD		Person with diabetes and hypertension	People with diabetes are more prone to depression	Depression due to loss of family/livelihood in patients with hypertension PTSD due to exposure to violence in a person with asthma

Underlying issues: humanitarian context, etc.

### What kind of interventions and models of care are required to ensure continuum and continuity of care?

Based on answers to questions one to three, (assessment of existing health structures, identification of priority diseases and target population), interventions can be developed and implemented in relation to screening, prevention, treatment, and rehabilitation. (Table 3).

**Table 3:** Level of crisis and type of intervention

	Emerging crisis	Acute crisis	Chronic crisis	Post crisis
Screening	No			
Prevention	Nutrition			Nutrition, healthy lifestyle promotion through the community, schools.
Treatment	Care for life-threatening complications, treatment of symptomatic conditions and complications, provision of treatment for persons already diagnosed and treated, education in self-care, detection and treatment of complications			
Rehabilitation	Physical rehabilitation	No	Physical rehabilitation, social support, support groups, surgical procedures…	

As stated by Evashwick, “continuum of care is a concept involving an integrated system of care that guides and tracks patients over time through a comprehensive array of health services spanning all levels of intensity of care” [46]. On the other hand, continuity of care relates to provision of quality care over time. The Sphere guidelines state that people with NCDs should be stabilized clinically and that “maintenance therapy” should be the humanitarian response [47]. The ideal model of care is NCD care integrated into existing primary health care, (PHC), facilities with simplification of protocols, provision of medicines and tools and task shifting, as done in HIV/AIDS programmes [48]. An integrated, multi and interdisciplinary approach should be chosen, involving community leaders and influential persons.

A robust system for registration and follow up for all patients with NCDs is required to ensure continuum of care. As proposed for crisis due to natural disasters, questionnaires integrated into medical records would allow identification of patients with pre-existing NCDs thus improving their management [17].

Patient records stored at health facilities, together with a summary of medical information carried by patients, would allow essential medical information to be available at the place where decisions are to be taken and insure quality and continuum of care [33]. These files need to outline current and former medical problems with co-morbidities, allergies, medications, diet and medical examinations, as well as onset of complications.

Adherence is vital in care for NCDs. Therefore, patient education to facilitate self-care as well as early detection and treatment of complications should be offered at every medical visit [49]. Culturally adapted “survival kits” of self-care with key messages for patients, such as the “five minute education kit for diabetes” [50] for health care providers and patients, empowers patients and assists health care providers to the teach most efficient strategies, such as management and prevention of hypoglycaemia. This is even more important in fragile environments where access to health care is challenging and patients may migrate and consult only in cases of complication.

Scope of provided interventions depends on crisis level. In emergencies, a comprehensive approach covering the whole spectrum of continuum of care may not be feasible. The priorities of managing NCDs in an acute crisis are to provide essential care for symptomatic conditions, to avoid discontinuation of treatment, to ensure access to essential diagnostic equipment and to establish referral mechanisms for conditions that cannot be managed at PHC level [3, 51].

First interventions include the provision of treatment with generic medicines at health facilities as done by the ICRC in Yemen and Libya, during the waiting period for the situation to improve so as to extend the services provided. Ideally medicines should carry simplified instructions, like the above mentioned “survival kits” on patient education. Treatment should be based on what existed before the crisis and adapted to the type of crisis. The level of care provision needs to be decided, (e.g. cancer treatment vs palliative care), the limits clearly stated and care integrated into existing health structures, (see ethical issues below).

Access to essential diagnostic equipment and medicines for persons previously identified with NCDs and risk factors have to be made available during a chronic crisis [8]. This is the case, for example, in ICRC supported PHC clinics in Lebanon. The screening for previously undetected NCDs is not recommended at any stage of an emergency unless it is part of the care of patients with a known NCD, (e.g. checking blood pressure in a diabetic patient, see ethical issues below).

Although preventive activities are challenging in humanitarian emergencies, a series of interventions are possible, such as provision of adequate food supplies in order to prevent malnutrition and chronic health problems [21, 52, 53] and patient education at all levels of care by various health care workers. Post-crisis primary prevention, such as the promotion of healthy lifestyles through community mechanisms [54] or the promotion of physical exercise should also be considered [55].

Finally, physical rehabilitation can be started after the acute phase of crisis. Physical rehabilitation therapists can, in addition to physical activity counselling, integrate other healthy lifestyle messages. This can build upon the vast experience that humanitarian organizations have in dealing with those wounded by war or natural disasters.

### Which algorithms or guidelines to use for the management of NCDs?

Ager et al. [56] highlighted the need for research in the field of NCDs in emergencies to identify appropriate tools and guidelines. Simplified protocols, guidelines and tools are urgently needed for the management of NCDs in humanitarian emergencies [57]. When available, existing national guidelines have to be followed for diagnosis, treatment, follow-up care, patient education and referral. Otherwise, the use of validated guidelines from the WHO, Sphere and humanitarian organizations, such as those developed by MSF or Primary Care International, (PCI), for management of diabetes and cardiovascular diseases, could be considered [47, 58–64]. Guidelines from high-income countries may need adaptation of tools, diagnostic tests, equipment and medication to the specificities of LMICs and emergency settings. Basic medical files are necessary for patient management. Availability of basic tests such as urine tests, blood tests for glucose, and medical equipment, (e.g. sphygmomanometer, weighing scale, tuning fork), need to be taken into account when selecting algorithms and guidelines [26]. Point of care tests, (e.g.HbA1c, glucose, and creatinine) [38], for health providers with previous experience in using them, facilitate patient management, in particular, in settings without timely access to laboratory services. Schroeder et al. [65] state that “listed tests should be reasonably available for people who need them, whether in form of point of care tests in physicians’ offices and pharmacies or as high complexity tests in reference laboratories.” Nevertheless, in emergencies tests available have to be prioritised along with care priorities.

### What medication to be integrated into the basic essential drug list for NCDs?

Care of NCDs in emergency settings should guarantee access to essential therapies to reduce morbidity and mortality due to acute complications or exacerbation of chronic health condition [8, 47]*.* The WHO model list of essential medicines should serve as reference for NCD care [21]. Therefore, NCD medicines should be selected from that list and made available and affordable to patients. Some patients in conflict zones may be used to more expensive newer drugs, and consider generic drugs as unacceptable. Nevertheless, in emergencies a public health approach has to be chosen regarding resource allocation. Training of health professionals in both rational prescriptions and how to deal with such situations, as well as patient education, are essential to address this issue [66].

It is not generally recommended to introduce new therapeutic regimens for the management of chronic health conditions during the relief effort, especially if the regimen is unlikely to be continued after the emergency phase. Therefore, medicines need also to be aligned with the MoH essential medicine list and protocols [47]. In purchasing essential medicines, the quality of drugs must be guaranteed, and “humanitarian pricing” by pharmaceutical firms should be explored. When selecting essential medicines, potential co-morbidity of different NCDs in the same patient needs to be considered, with a special focus on drug interactions and contra-indications. This should be included in any clinical guideline developed.

The WHO is currently testing and implementing a new emergency health kit for NCDs in the Middle East. The kit contains essential medicines for the management of cardiovascular diseases including hypertension, diabetes mellitus, chronic respiratory diseases and management of some mental health and neurological conditions [67]. In addition, inclusion of NCD-related essential medicines for the treatment of complications is considered in the 2016 revision of the composition of the Interagency Emergency Health Kit (IEHK) [68, 69]. Medicines provided should not only treat exacerbations but also guarantee a continuum of care with medicines for on-going treatment [51].

Supply and cold chains have to be ensured and medicines made available to health facilities close to the patients. For the delivery of medicines, the access of patients to health care facilities and pharmacies has to be assessed as well as whether or not patients are free to choose the health facility or have to go to the facility of their catchment area. Continuum of care would require the follow-up and delivery of medicines at the same health facility, or the transfer of patient data and register – as well as informing the patients where to find the medicines.

### What are the ethical issues related to care for NCDs?

A public health approach has to be chosen regarding resource allocation, level of care and prioritisation of NCDs. For example, cancer care with chemotherapy is rarely possible in humanitarian emergencies [36]. Ethical issues may relate to the sustainability of providing long-term cancer care in constraint environments and by humanitarian agencies that have short-term mandates [57]. Similarly, haemodialysis might require a level of medical complexity, long-term commitment, and financial resources that would not be within reach of most agencies involved in relief response.

NCDs need continuity of care and reliance on therapeutic adherence which is reinforced by patient education. Continuity in access to drugs is essential since shortages may put the patient at risk [20] and interruption of treatment may cause rebound effects, worsening patients’ conditions [70]*.* Therefore, collaboration with local health authorities and integration of care into existing health structures is very important. However, in acute emergencies the establishment of efficient immediate response, integration of care is not always possible. This is also the case if the local health system is disrupted. Withdrawal from support of international organizations has to be planned with adequate handover to local authorities and/or relevant health actors at a later stage when the situation is more stable.

Humanitarian actors are currently exploring the use of electronic patient files and registers. These technologies require training of health professionals on their use, sending technicians on-site for maintenance and making electricity available with back-up systems. This may divert resources from providing treatment in emergencies. In chronic crisis some positive experiences have been reported by UNWRA with easier access to patient records, better data management and follow-up of patients [71, 72]. Connectivity is a critical element for mobile technology. In emergencies, states may temporarily shut down access to Internet. Therefore, applications need to be able to temporarily store data locally and synchronize when having access to the web to communicate [73]. Data protection and confidentiality of medical information needs to be taken into account if considering storing data in the Cloud. Medical data is sensitive and has to be protected against unauthorized disclosure or use. This requires safe storage and transmission, and other measures in line with data protection principles [74]*.* Use of applications on tablets and phones, would depend on what was available before the crisis and patient literacy, so as not to introduce technology which is not sustainable and appropriate for patient care.

As stated in the section regarding model of care, screening and active case finding should not be included as part of interventions in emergencies. Screening causes ethical dilemmas as once a case has been diagnosed, access to care and treatment needs to be provided, overburdening already stretched health systems [8]. Screening also raises the challenge of tackling false positive cases. However, in selected programmes, where sufficient appropriate treatment is available, active case finding could be considered during medical consultations in high-risk patients, (e.g. checking blood pressure in a diabetic patient or in pregnant women in all settings).

### How to be accountable to patients?

Patient-centred care answers the needs of patients with chronic health problems. Whereas in an acute crisis the provision of treatment is the priority, (e.g. provision of insulin for people with type 1 diabetes in Yemen by the ICRC, or evacuation of patients to more stable parts of the country [24]), in chronic crisis, self-care and challenges of living with a NCD and potential mental health problems need to be addressed at each consultation, and solutions found in collaboration with patients, families, communities and health professionals. Comprehensive responses are initiated by MSF in Jordan including patient groups, for health education, home visits and mental health counselling [37] and by UNWRA with family health teams in their health structures [75]*.* When designing an intervention, the cultural aspects related to the NCD have to be taken into account. Patient education has to be adapted to the patient’s level of understanding with appropriate language and tools (e.g. cooking book developed with patients and families by UNWRA). Families and communities’ support for patients with NCDs are a central part of chronic care models. Patient accountability also means not screening for conditions for which no treatment is available. Therefore, in emergencies, the focus should be on patients with complications and those with known health problems under treatment. As triage is required, not only for surgical but also for medical problems, the level of staff education and experience plays a major role in quality of NCD care. Major decisions, such as abstention of care or amputation, warrant a second opinion and should be in line with local standards.

As for all medical interventions, the quality and availability of provided drugs, and data protection need to be guaranteed, and validated guidelines applied.

### How to monitor the intervention?

Monitoring is also part of the accountability to beneficiaries, and also the accountability to the organization implementing the intervention and its donors. Indicators should be easy to collect and not overburden health workers and divert resources. Interventions on different levels, from the GAP [21], to national and local health facilities and patients can be monitored. However in emergencies, the use of indicators for prevention and control of NCDs according to the WHO GAP is challenging, as primary prevention is only feasible in post-crisis situations. Nevertheless, indicators to monitor access and availability of care for patients with NCDs are proposed by the United Nations (UN) interagency taskforce [8]. At the health facility level, pre-identified indicators such as number of patients per month with their primary outcome status, (alive, dead, default, drop out, transfer), vital signs and laboratory parameters specific to each NCD and complication rate can be used to assess accessibility, continuum and quality of care. These data combined with monthly use of medication and devices, facilitates adaptation of interventions and the planning of resources, (medication, devices, human resources and training). Innovative methods to estimate the efficacy of interventions in challenging environments where no randomized controlled trials are feasible, could be tested, (such as interrupted time series analysis) [76]*.* Patient files are used to monitor patients’ individual health and to adapt treatments. Indicators, integrated into patient files, facilitate their extraction and use for monitoring and evaluation. Monitoring via patient tracking using electronic patient records [26, 57, 77] could be considered if data protection is guaranteed.

### What to do beyond the provision of health services for “classical” NCDs?

Beyond the provision of health services for “classical NCDs” as outlined by the WHO the following issues should be included in humanitarian responses to crisis:

- Mental health problems face similar challenges in continuum of care as other NCDs with a rising burden of mental health conditions during a crisis. Mental health issues affect six times more people than those wounded in a conflict and the prevalence of mental health problems increases after a disaster [15, 78]. In addition, mental illness has a negative impact on patients’ self-management of their NCD and on their NCD outcome. Interventions in the field of mental health should address stigma, be culturally appropriate, respect the primum non-nocere –“do no harm”- approach and ensure continuous “supportive supervision”. The WHO addresses mental health in the Mental Health Gap Action Programme (mhGAP), together with care for epilepsy, as these conditions are not included in the “classical” NCD response [64]. Drugs for the management of psychosis, depression and epilepsy are part of WHO’s NCD medicine kit [67] which is currently tested in the Middle East, as well as integrated in MSF [79], UNHCR [80] and ICRC essential list of medicines. Therefore, mental health interventions should be considered along with the management of the WHO prioritised NCDs in emergencies.
- One way of supporting first line responders in a crisis [81], as done by the ICRC in Syria, has been the improvement of patient care through increasing the resilience of carers, by addressing their skills in basic psychological support and avoiding migration out of the emergency setting.
- Appropriate nutrition is part of the management of diabetes, hypertension and cardiovascular diseases. In a crisis, access to food is frequently erratic and affects the outcome of patients. Therefore, humanitarian actors should advocate to food providers the supply of appropriate types and amount of food. In addition, poor nutrition in children is a risk factor for the development of future chronic health problems [21, 52, 53]. Therefore, access to food is an integral part in interventions for NCDs [82]*.*
- Safe access to health facilities in fragile environments is key to follow-up patients over time and across health care levels. All actors and stakeholders across states need to collaborate so as to guarantee the protection and respect of patients, health personnel, infrastructure, medical goods and transportation services [83]. Unimpeded access of patients to existing services is also essential.
- Research and evidence on how to effectively address care for NCDs in emergencies is lacking [22, 56]*.* However, advocating for NCD research in a crisis is challenging, due to the nature of an emergency response, urgency of other needs in addition to NCDs being an artificial construct constituted by different diseases. Diseases for which humanitarian agencies provide care range from diabetes, cardiovascular and chronic respiratory diseases, to epilepsy, hypothyroidism, thalassemia, sickle cell anaemia, mental health problems and chronic kidney failure. Therefore, to advocate for NCD care and research in humanitarian settings, a disease, like diabetes type 1 and the need to ensure insulin supply, can be chosen as “the show case”, with which to build a case.
- Solid and sustainable interventions for NCDs are a challenge in humanitarian emergencies. Regular partnership meetings with humanitarian actors and academia lead by UNHCR allow exchange and aligning of practices [84]. A coordinated and multi-stakeholder approach – ranging from governments to humanitarian agencies, research and academic institutions with concrete engagements and advocacy would allow better responses to the needs of affected populations [22, 32, 85, 86].

## Conclusions

Addressing the global burden of NCDs in emergencies is a challenging task. When planning a relief operation in a country, humanitarian actors could consider 10 key questions to help guiding interventions. The choice of intervention depends on the nature and impact of the crisis, the existing health system, (previous and current), available resources - including new actors - and access of patients to healthcare. Prioritisation is needed with regard to which NCDs to address based on the burden of disease in each setting and gaps in the provision of health care. Interventions should be context specific and tailored to the target population, (e.g. interventions for a refugee camp in Kenya vs refugees living in urban settings in Lebanon). NCDs are chronic in nature and demand long-term commitment beyond acute care provision. The focus of interventions in acute crises is management of acute conditions and patients’ access to treatment, whereas in stable settings, the management of NCDs has a strong focus on preventive activities, which are not feasible in crises. A patient-centred approach is critical. This includes educating patients on self-management, provision of a stock of essential medicines and information on where to find additional care. Interventions need to be monitored using simple indicators to assess their effectiveness. Safe access to care and support to health staff are required for solid and sustained health interventions in emergencies. Finally, in order to address NCDs in a comprehensive way in humanitarian emergencies, a multi-stakeholder approach that includes health care providers, governments, humanitarian agencies and academic institutions is required.

## Abbreviations

GAP: Global Action Plan
ICRC: International Committee of the Red Cross
IEHK: Interagency Emergency Health Kit
LMIC: Low and middle-income countries
mhGAP: Mental Health Gap Action Programme
MoH: Ministry of Health
MSF: Médecins Sans Frontières
NCD: Non-communicable diseases
PCI: Primary Care International
PHC: Primary Health Care
RAPIA: Rapid assessment protocols for insulin access
SARA: Service Availability and Readiness Assessment
UN: United Nations
UNHCR: United Nations High Commissioner for Refugees
UNWRA: United Nations Relief and Works Agency for Palestine Refugees in the Near East
WHO: World Health Organization

## References

[1] World Health Organization (WHO). World Health Statistics 2016: Monitoring health for the SDGs. World Health Organization. 2016.

[2] Marshall S.J. Developing countries face double burden of disease. Bull WHO. 2004;82:7:556. PMC2622909 15500291

[3] International Committee of the Red Cross (ICRC). Non communicable diseases in health interventions, guiding principles. International Committee of the Red Cross. 2012.

[4] Halford G. Prevalence of Diabetes in ICRC Supported Physical Rehabilitation Centers. International Committee of the Red Cross. 2016;

[5] World Health Organization (WHO). Environmental health in emergencies and disasters: a practical guide. World Health Organization. 2002.

[6] International Committee of the Red Cross (ICRC). Les types de crise. Assistance. Les types de crise et illustration du cycle à travers un exemple. Module préparatoire. Programme d’intégration. International Committee of the Red Cross. 2009. doi:10.1371/currents.dis.53e08b951d59ff913ab8b9bb51c4d0de.

[7] Perrin P. War and Public Health. In Handbook on War and Public Health. International Committee of the Red Cross. 1996.

[8] United Nations Interagency Task-Force on the Prevention and Control of Non-communicable Diseases. Non communicable diseases in emergencies. WHO/NMH/NVI16.2. 2016.

[9] Demaio A, Jamieson J, Horn R, de Courten M, Tellier S. Non-Communicable Diseases in Emergencies: A Call to Action. PLoS Curr Disasters. 2013; 10.1371/currents.dis.53e08b951d59ff913ab8b9bb51c4d0de PMC3775888 24056956

[10] Nickerson JW, Hatcher-Roberts J, Adams O, Attaran A, Tugwell P. Assessments of health services availability in humanitarian emergencies: a review of assessments in Haiti and Sudan using a health systems approach. Conf Health 2015; DOI 10.1186/s13031-015-0045-6. 10.1186/s13031-015-0045-6 PMC4477304 26106443

[11] Hayman KG, Sharma D, Wardlow RD, Singh S (2015). Burden of cardiovascular morbidity and mortality following humanitarian emergencies: a systematic literature review. Prehosp Disaster Med.

[12] Pan American Health Organization (PAHO) (2000). General Effects of Disasters on Health. Natural Disasters: Protecting the Publics Health.

[13] Schreeb J, Riddez L, Samnegård H, Rosling H. Foreign Field Hospitals in the Recent Sudden-Onset Disasters in Iran, Haiti, Indonesia, and Pakistan. Prehosp Disaster Med 2008; doi:10.1017/S1049023X00005768. 10.1017/s1049023x00005768 18557294

[14] Guha-Sapir D, van Panhuis WG, Lagoutte J (2007). Short communication: patterns of chronic and acute diseases after natural disasters - a study from the International Committee of the Red Cross field hospital in Banda Aceh after the 2004 Indian Ocean tsunami. Tropical Med Int Health.

[15] Redwood-Campbell LJ, Riddez L (2006). Post-tsunami medical care: health problems encountered in the International Committee of the Red Cross Hospital in Banda Aceh, Indonesia. Prehosp Disaster Med.

[16] Mobula LM, Fisher ML, Lau N, Estelle A, Wood T, William Plyler W. Prevalence of Hypertension among Patients Attending Mobile Medical Clinics in the Philippines after Typhoon Haiyan Version 1. PLoS Curr. 2016;8: ecurrents.dis.5aaeb105e840c72370e8e688835882ce. Published online 2016 December 20. 10.1371/currents.dis.5aaeb105e840c72370e8e688835882ce PMC5325669 28286697

[17] Sharma AJ (2008). Chronic Disease and Related Conditions at Emergency Treatment Facilities in the New Orleans Area After Hurricane Katrina. Disaster Med. Public Health Prep.

[18] Zwi A, Ugalde A (1989). Towards an epidemiology of political violence in the third world. Soc Sci Med.

[19] Besançon S, Fall I-S, Doré M, Sidibé A, Hagon O, Chappuis F, Beran D (2015). Diabetes in an emergency context: the Malian case study. Confl Heal.

[20] Spiegel PB, Checchi F, Colombo S, Paik E (2010). Health-care needs of people affected by conflict: future trends and changing frameworks. Lancet.

[21] World Health Organization (WHO). Global Action Plan for the Prevention and Control of Noncommunicable Diseases 2013–2020 - Revised draft (Version dated 11 February 2013). Geneva. World Health Organization. 2013.

[22] Maurer P. Non-communicable diseases in fragile contexts, presented at the Side event on non-communicable diseases United Nations General Assembly 2016. NYC, USA. 2016.

[23] Doocy S (2016). Health Service Utilization among Syrian Refugees with Chronic Health Conditions in Jordan. PLoS One.

[24] Besançon S. Diabetes in Emergency Context in West Africa: The Example of Mali. presented at the International Conference on Refugees and Diabetes, Dead Sea, Jordan. 2017.

[25] World Health Organization (WHO). How to Investigate Access to Care for Chronic Non-communicable Diseases in Low- and Middle-income Countries. A Survey Manual Based on a Rapid Assessment Protocol. Draft for Field Testing. World Health Organization. 2012.

[26] Mendis S (2012). Gaps in capacity in primary care in low resource settings for implementation of essential non-communicable disease interventions. Int J Hypertens.

[27] International Committee of the Red Cross (ICRC). Contingency plan - Health, Questions and Answers. International Committee of the Red Cross. 2011.

[28] Ebola in Context: Understanding Transmission, Response and Control. Presented at the Online Course - FutureLearn, London, LSHTM. 2015. https://www.futurelearn.com/courses/ebola-in-context.

[29] Muga F (2015). Ebola – Not just another epidemic, Pac. J Med Sci.

[30] Haskew C, Spiegel P, Tomczyk B, Cornier N, Hering H (2010). A standardized health info system for refugee settings: rationale, challenges and the way forward. Bull WHO.

[31] Primary Care International. Strengthening family medicine woldwide. https://pci-360.com/#24-2.

[32] Jobanputra K, Boulle P, Roberts B, Perel P. Three Steps to Improve Management of Non-communicable Diseases in Humanitarian Crises. PLOS Medicine. 2016; doi:10.1371/journal.pmed.1002180. 10.1371/journal.pmed.1002180 PMC5100924 27824879

[33] Slama S. et al. Care of non-communicable diseases in emergencies. Lancet. 2016; doi:10.1016/S0140-6736(16)31404-0.

[34] Boulle P. Monitoring and evaluation of NCD programmes in humanitarian settings - challenges and opportunities; presented at the 5th partner meeting: Management of NCDs in humanitarian settings, Geneva. 14-Nov-2016.

[35] Beran D. The Rapid Assessment Protocol for Insulin Access (RAPIA): research for action on access to diabetes care. MERA: Diabetes Int. 2009;17(1):4–8.

[36] Spiegel P, Khalifa A, and Mateen FJ. Cancer in Refugees in Jordan and Syria between 2009 and 2012: Challenges and the Way Forward in Humanitarian Emergencies. Lancet Oncology 2014; doi:10.1016/S1470-2045(14)70067-1. 10.1016/S1470-2045(14)70067-1 24872112

[37] Garret P. Challenges and Experiences in Delivering Direct Diabetes Care to Refugees. Presented at the International Conference on Refugees and Diabetes, Dead Sea, Jordan. 2017.

[38] Médecins Sans Frontières (MSF). Innovation unit OCG. Chronic noncommunicable diseases in OCG – Position paper: Médecins Sans Frontières. 2016.

[39] Shahin Y, Kapur A, Seita A. Diabetes care in refugee camps: the experience of UNWRA. Diabetes Res Clin Pract. 2015. 10.1016/j.diabres.2015.01.05. 10.1016/j.diabres.2015.01.035 25680680

[40] Khader A, Farajallah L, Shahin Y, Hababeh M, Abu-Zayed I, Zachariah R, Kochi A, Kapur A, Harries AD, Shaikh I, Seita A (2014). Hypertension and treatment outcomes in Palestine refugees in United Nations Relief and Works Agency primary health care clinics in Jordan. Tropical Med Int Health.

[41] Jadresh D, Woodman M. UNHCR diabetes care provision to refugees in the MENA region. Presented at the International Conference on Refugees and Diabetes, Dead Sea, Jordan. 2017.

[42] Kapur A. Background Paper for the International Conference on Refugees and Diabetes. UNWRA, United Nations Relief and Works Agency for Palestine Refugees in the Near East. 2017.

[43] Boulle P. Experience from conflict settings – burden of disease and programmatic challenges. Presented at the Symposium on NCDs in humanitarian settings. London; LSHTM. 2016.

[44] Doocy S, Lyles E, Hanquart B, Woodman M, LHAS Study Team (2016). Prevalence, care-seeking, and health service utilization for non-communicable diseases among Syrian refugees and host communities in Lebanon. Confl Heal.

[45] Nezar I, Taib A. Mobile clinic to reach the unreachable; presented at the Geneva Health Forum, Geneva. 2016.

[46] Evashwick C (1989). Creating the continuum of care. Health Matrix.

[47] The Sphere Project. Sphere handbook: Essential health services – non communicable diseases. Geneva. 2011.

[48] Edwards JK, Kosgei RJ, Sobry A, Vandenbulcke A, Vakil SN, Reid T (2015). HIV with non-communicable diseases in primary care in Kibera, Nairobi, Kenya: characteristics and outcomes 2010-2013. Trans R Soc Trop Med Hyg.

[49] World Health Organization (WHO). World Health Day 2016: WHO calls for global action to halt rise in and improve care for people with diabetes. World Health Org 2016.

[50] Diabetes Education Study Group of the European Association for the Study of Diabetes (1995). Survival Kit: The Five-Minute Education Kit. A Document for Health Care Providers and Patients. Diabet Med.

[51] Aebischer Perone S, Beran D (2017). Modifying the Interagency Emergency Health Kit to Include Treatment for Non-Communicable Diseases in Natural Disasters and Complex Emergencies: The Missing Clinical, Operational and Humanitarian Perspectives. BMJ Global Health.

[52] Gluckmann PD, Hanson MA, Cooper C (2008). Effect of in utero and early life conditions on adult health and disease. N Engl J Med.

[53] Ravelli Ac, van der Meulen JH, Michels RP, et al. Glucose tolerance in adults after prenatal exposure to famine. Lancet 1998; 351:173e7. 10.1016/s0140-6736(97)07244-9 9449872

[54] International Federation of Red Cross and Red Crescent Societies (IFRC). Healthy lifestyle community. 2016. [Online]. Available: http://www.ifrc.org/en/what-we-do/health/diseases/noncommunicable-diseases/ncds-toolkit/.

[55] Dejgaard A. Model of care in diabetic management. Presented at the Global Partnerships for Humanitarian Impact and Innovation meeting. Session on non-communicable diseases. How to better manage a patient with NCD through a holistic model of care and how the new technology could contribute? Lausanne. 2016.

[56] Ager A, Burnham G, Checchi F (2014). Strengthening the evidence base for health programming in humanitarian crises. Science.

[57] Ruby A, Knight A, Perel P, Blanchet K, Roberts B. The effectiveness of interventions for non-communicable diseases in humanitarian crises: A systematic review. PLOS ONE. 2015 ; doi: 10.1371/journal.pone.0138303. 10.1371/journal.pone.0138303 PMC4583445 26406317

[58] Médecins Sans Frontières (MSF). Innovation unit OCG. Testing and clinical management of diabetes in MSF settings, Field manual. Geneva, Switzerland: Médecins Sans Frontières. 2014. msf.innovationportal.eu

[59] Médecins Sans Frontières (MSF). Innovation unit OCG. Integrated clinical pathway for patients at high cardiovascular risk. Geneva, Switzerland: Médecins Sans Frontières. 2014. msf.innovationportal.eu

[60] World Health Organization (WHO). Package of Essential Non-communicable (PEN) disease interventions for primary health care in low-resource settings. World Health Organization. 2010.

[61] World Health Organization (WHO). Package of Essential Non-communicable (PEN) Disease Interventions for Primary Health Care in Low-Resource Settings. World Health Organization, Geneva. 2013.

[62] Primary Care International (PCI). NCD Field Guide Based on WHO Essential Medicines List, Guide on Asthma, COPD, Diabetes, Hypertension (with Severe and Hypertension in Pregnancy) and CVD Secondary Prevention. United Nations High Commissioner for Refugees (UNHCR), 2016.

[63] Jobanputra K. Non-Communicable Diseases - Programmatic and Clinical Guidelines. Médecins Sans Frontières, 2016. fieldresearch.msf.org

[64] World Health Organization (WHO). Mental health Gap Action Programme Intervention guide. mhGAP version 2.0, World Health Organization. 2016. 27786430

[65] Schroeder LF, Guarner J, Elbireer A, Castle PE, Amukele TK. Time for a Model List of Essential Diagnostics. N Engl J Med. 2016; doi: 10.1056/NEJMp1602825. 10.1056/NEJMp1602825 27355530

[66] Persad GC, Emanuel EJ. The ethics of expanding access to cheaper, less effective treatments. Lancet. 2016; doi: 10.1016/S0140-6736(15)01025-9. 10.1016/S0140-6736(15)01025-9 27108231

[67] World Health Organization (WHO). WHO revised Non-communicable Diseases (NCD) Emergency Kit. WHO EMRO. 2016.

[68] World Health Organization (WHO) (2011). The Interagency Emergency Health Kit 2011.

[69] Tonelli M, Wiebe N, Nadler B, Darzi A, Rasheed S. Modifying the Interagency Emergency Health kit to include treatment for non-communicable diseases in natural disasters and complex emergencies. BMJ Glob Health. 2016;1 doi:10.1136/bmjgh-2016-000128. 10.1136/bmjgh-2016-000128 PMC5321368 28588970

[70] Regard S (2011). Non communicable diseases: what’s next? International Committee of the Red Cross.

[71] United Nations Relief and Works Agency for Palestine Refugees in the Near East, (UNWRA). Technology Revolutionizes Care in UNWRA Health Clinics. United Nations Relief and Works Agency for Palestine Refugees in the Near East. 2015. https://www.unrwa.org/newsroom/features/technology-revolutionizes-care-unrwa-health-clinics.

[72] Khader A, Ballout G, Shahin Y, Hababeh M, Farajallah L, Zeidan W, Abu-Zayed I, Kochi A, Harries AD, Zachariah R, Kapur A, Shaikh I, Seita A (2013). Diabetes mellitus and treatment outcomes in Palestine refugees in UNRWA primary health care clinics in Jordan. Public Health Action.

[73] Eppings P. What the new mobile technology could bring for better management of the patients in NCDs, presented at the Global Partnerships for Humanitarian Impact and Innovation meeting. Session on non-communicable diseases. How to better manage a patient with NCD through a holistic model of care and how the new technology could contribute? Lausanne. 2016.

[74] Barber B, Scholes M. Reflections On the Development of Medical Informatics. Acta Inform Med. 2014; doi: 10.5455/aim.2014.22.18-24. 10.5455/aim.2014.22.18-24 PMC3948183 24648616

[75] Santoro A. Abu-Rmeileh N., Khader A., Seita A., McKee M. Primary healthcare reform in the United Nations Relief and Works Agency for Palestine Refugees in the Near East. Short communication. EMHJ. 2016;22(6):417–421. ISSN 1020–3397. Downloaded from http://researchonline.lshtm.ac.uk/2997173/1/EMHJ_2016_22_06_417_421.pdf. 10.26719/2016.22.6.417 27686983

[76] Prieto D. Innovative methods in assessment /surveys for challenging settings. Presented at the Symposium on NCDs in humanitarian settings, London, LSHTM. 2016.

[77] Khader A, Farajallah L, Shahin Y (2012). Cohort Monitoring of Persons with Hypertension: An Illustrated Example from a Primary Healthcare Clinic for Palestine Refugees in Jordan: Cohort Reporting for Hypertension. TMIH.

[78] van Ommeren M, Saraceno B (2005). Aid after disasters. Needs a long term public mental health perspective. BMJ.

[79] Pilon, Sophie. Essential Drugs. Practical Guide Intended for Physicians, Nurses and Medical Auxiliaries. Reference Books MSF. Médecins Sans Frontières, 2016. http://refbooks.msf.org/msf_docs/en/essential_drugs/ed_en.pdf.

[80] United Nations High Commissioner for Refugees (UNHCR). UNHCR Essential Medicines List Valid from October 2016 to October 2018. United Nations High Commissioner for Refugees. 2016.

[81] International Committee of the Red Cross (ICRC). Helping the helpers- why does psychosocial support matter? International Committee of the Red Cross. 2015.

[82] Grijalva-Eternon et al. 2012 The Double Burden of Obesity and Malnutrition in a Protracted Emergency Setting: A Cross-Sectional Study of Western Sahara Refugees. PLoS Med. 2012; 10.1371/journal.pmed.1001320 10.1371/journal.pmed.1001320 PMC3462761 23055833

[83] International Committee of the Red Cross (ICRC). Protecting health care, key recommendations. International Committee of the Red Cross. 2016.

[84] United Nations High Commissioner for Refugees. 5th partner meeting: Management of NCDs in humanitarian settings, Geneva. 2016.

[85] Martinez E. NCDs in refugees and migrants – the hidden crisis. World Cancer Research Fund International. 2016; http://www.wcrf.org/int/blog/articles/2016/09/ncds-refugees-and-migrants-hidden-crisis.

[86] United Nations Relief and Works Agency for Palestine Refugees in the Near East (UNWRA), World diabetic foundation. (WDF). Dead Sea Declaration and Call to Action on Refugees and Diabetes. Presented at the International Conference on Refugees and Diabetes, Dead Sea, Jordan. 2017.

[87] Beran D, Sartorius N, Holt RIG, Maj M (2015). Difficulties Facing the Provision of Care for Multimorbidity in Low-Income Countries. Comorbidity of Mental and Physical Disorders.

